# Letter from Galva, Ill.

**Published:** 1883-09

**Authors:** J. P. Lytle

**Affiliations:** Galva, Ill.


### Domestic Correspondence.

### ARTICLE XII.

GALVA, ILL., June 28, 1883.

EDITORS CHICAGO MEDICAL JOURNAL AND EXAMINER.

*Dear Sir:*—Several years ago I saw a suggestion in one of the medical journals that a spray of dilute soctic acid would dissolve pseudo membrane of croup.

I concluded that if it would do so, it would also dissolve the membraneous deposit of diphtheria. Since then I have used it in a number of cases with the happiest results. Where the patient is old enough to permit a thorough spraying of the throat I have not failed to remove the deposit with two or three applications, sometimes with one. May 4, 1883, 4 P.M., was called to see young Mr B., aged sixteen. Face flushed; pulse quick; temperature 102; throat very painful, and deglutition very difficult. Upon examining his throat, I discovered a gray, gelatinous membrane fully one-eighth of an inch thick, covering the entire tonsil, and extending over the soft palate of left side; right inflamed and swollen, but no deposits upon it. I sprayed the throat with the following solution. Acid soctic concreet, gtts xxx, aqua ℥i, and had the gratification of seeing the membrane entirely disappear. I put him on general treatment, stating that I would call in the morning. I then found the left tonsil and palate clear; but the right tonsil and palate had upon it a deposit fully as extensive as the left had upon it the night before. I again used the spray, and had the satisfaction of seeing the throat clear of deposit when I was through. I continued the general treatment, and left a solution acid soctic gtts x, agua ℥i, to be used as a gargle. He made a rapid recovery, and there was no more deposit of membrane. To those of your readers who have not used the acid in this way they will find it worthy of trial. I use a double-bulb rubber atomizer with rubber tip.

Yours very respectfully,

J. P. LYTLE, M.D.

